# Gap analysis on hospitalized health service utilization in floating population covered by different medical insurances ----- case study from Jiangsu Province, China

**DOI:** 10.1186/s12939-019-0992-4

**Published:** 2019-06-10

**Authors:** Xinzhao Cai, Fan Yang, Ying Bian

**Affiliations:** 1 State Key Laboratory of Quality Research in Chinese Medicine, Institute of Chinese Medical Sciences, University of Macau, Macau, China; 2 School of Health Policy & Management, Nanjing Medical University, Nanjing, Jiangsu China

**Keywords:** Floating population, Inpatient health utilization, Health insurance coverage, Jiangsu China

## Abstract

**Objective:**

By analyzing the gap of hospitalization service among floating population covered by different medical insurance in Jiangsu Province, this paper aimed to understand the current situation of hospitalized health service utilization (HHSU) among floating population, and to provide policy suggestions for improving HHSU of floating population with different health insurance.

**Methods:**

The data of this study were obtained from “the National Dynamic Monitoring Survey of Floating Population in 2014”. A total of 12,000 samples of floating population in Jiangsu Province were selected. 57.15% for men and 42.85% for women; 46.95% for those under 30 years old, 39.67% for 30 to 45 years old, 13.38% for over the age of forty-five. Using descriptive statistical analysis, chi-square test, exploratory factor analysis, logistic regression and stepwise multiple linear regression, the paper analyzed the difference of HHSU of floating population with different medical insurance in 2014. This study divided basic medical insurance into 3 categories: MIUE (Medical Insurance of Urban Employee), other medical insurances (including new rural cooperative medical system and the medical insurance for urban residents) and no medical insurance.

**Results:**

The hospitalization rate of floating population with MIUE (89.95%) was higher than the rate of floating population with other medical insurances (74.76%) and the gap is 15.19%. It was also higher than the rate of floating population with no medical insurance (67.57%) and the gap is 22.38%. (chi-square = 24.958, *p* = 0.000). 15.34% of floating population with MIUE spent more than 1600 dollars during hospitalization. It was lower than floating population with other medical insurances (16.19%) and no medical insurance (21.62%). The gaps respectively were 0.85 and 6.28% (chi-square = 10.000, *p* = 0.040). There existed significant differences among hospitalization medical expenses that floating population with different basic medical insurances spent. (chi-square = 225.206, *p* = 0.000) The type of basic medical insurance had statistical significance on whether the patients were hospitalized (*p* = 0.003) and whether they were hospitalized (*p* = 0.014). Logistic regression analysis results showed that “Social structure” (Education, Hukou, Insurance status and Work status) were significantly associated with Should be hospitalized but not and “Education” were significantly associated with Inpatient facilities selection. The stepwise multiple linear regression results presented that “Demography” and “Floating area” influenced In-hospital medical cost and “Social structure” and “Gender” influenced Reimbursement of in-hospital medical cost.

**Conclusion:**

Medical insurance type affects the hospitalization health service utilization of floating population, including Should be hospitalized but not and Reimbursement of in-hospital medical cost.

## Bakground

There is over 260 million floating population in mainland China which is 19.5% of the total population. At the same time, the scale of floating population is constantly expanding, and it will exist as a special group for a long time [1, 2]. Floating population’s health have been threatened by lack of basic health care, unbalanced allocation of health resources and inequity of health services [3–5].

Affected by policies and economic factors, the supply of medical resources in cities is limited. Medical resources that originally belonged to the urban population need to be allocated to the floating population. Due to the existence of a large number of floating population, floating population and local population cannot obtain reasonable quantity and quality of health service [6, 7]. The problem of floating population’s hospitalized health service utilization has become more serious.

China’s floating population is mainly concentrated on well-developed regions. From 2005 to 2014, Jiangsu’s GDP ranked the top three among provincial administrative regions, and its per capita GDP ranked the top three for ten consecutive years. The total population of Jiangsu Province is 79.7 million in 2014. More than 17 million floating population had registered in Jiangsu by 2013. The health problem of floating population is also becoming the focus in the field of medical insurance.

China’s basic medical insurance covers a wide range of types, including medical insurance for urban employees (MIUE), medical insurance for urban residents (MIUR), new rural cooperative medical system (NCMS) and medical insurance for urban and rural residents (MIURR). The reimbursement modes of various insurances are different. It is difficult for floating population to get reimbursed for medical expenses in the places where they live without residence registration. In general, they expended too much manpower, material and financial resources to get inpatient health service.

## Objective

This study intends to understand the current situation of inpatient health service utilization of floating population by analyzing the gap in hospitalized health services under different medical insurance in Jiangsu Province, and analyze the current situation of inpatient health service utilization of floating population under urban employee medical insurance and other medical insurances and no medical insurance.

## Methods

### Sampling

The data of this study was obtained from “the National Internal Migrant Dynamic Monitoring Survey, 2014”. The survey focused on the health of the floating population across the country to find out the existing problems.

This survey adopted stratified and multi-stage sampling method and selected sample points in the areas in Jiangsu province where the floating population is concentrated in according to the random principle. It includes 13 prefecture-level cities including Nanjing, Wuxi, Suzhou, Changzhou, Zhenjiang, Nantong, Taizhou, Yangzhou, Lianyungang, Huai’an, Suqian, Yancheng and Xuzhou. In this study, all the floating population in Jiangsu province in the survey was selected as the research object, with a total of 12,000 people.

### Ethics approval and consent to participate

Not applicable; this was a secondary analysis of de-identified data. The “National Internal Migrant Dynamic Monitoring Survey, 2014” data is publicly available to authorized researchers who have been given permission by the National Population and Family Planning Commission, and written informed consents were obtained from all participants. Information regarding the approval and consent process for the 2014 National Dynamic Monitoring Survey on Migrants has been published previously [Department of Services and Management for Migrant Population of NHFPC (DSMMP) 2015 Report on China’s migrant population development. Beijing: China Population Publishing House; 2015.].

### Definitions in this study

Hukou: A rigid household registration system in China that serves as a domestic passport, which regulates population distribution and rural-to-urban migration [8–10]. Under the “Hukou” system, floating population cannot share most of the privileges as urban dwellers do, such as health insurance in local city.

Floating population: There is no unified and accepted definition of floating population at present [11]. “Floating population”, as defined by the authority, is a person who is detained in the place of residence because of work, life and other reasons but has not registered in this place [12–15]. In this study, floating population aged between 15 and 60 who have worked or lived in the residential area for more than one month without Hukou.

Should be hospitalized but not: Under the doctor’s diagnosis, someone should be hospitalized but ultimately do not choose to use inpatient health services due to certain reasons.

### Measures

This study measures the gap between different health insurances coverage in the utilization of in-hospital health services among floating population [16]; On this basis, descriptive statistical analysis, chi-square test, exploratory factor analysis, logistic regression and stepwise multiple linear regression were used to study the gap of the utilization of in-hospital health services among floating population under different insurances from the perspective of the impact of population characteristics on the hospitalized health service utilization.

### Statistical analysis

The basic medical insurance types were divided into 3 types: MIUE (Medical Insurance of Urban Employee), other medical insurances (including new rural cooperative medical system and the medical insurance for urban residents) and no medical insurance. Exploratory factor analysis was conducted to assess the dimensionality of influencing factors and reduce the number of variables. Logistic regression and stepwise multiple linear regression analysis were implied to explore the association between HHSU in floating population and its influencing factors. Besides, this study also used descriptive statistical analysis, one-way ANOVA, chi-square test. When *P* < 0.05, the difference was statistically significant. SPSS 20.0 statistical software and Microsoft Excel 2010 were used to analyze the data.

## Results

### Sample characteristics

Table 1 shows the result of descriptive statistics for variables used in the analyses by the type of medical insurance, as well as the overall statistics. Floating population under MIUE are older than others(*p* < 0.001). The majority of the sample was male (57.98%) and most of the participants were married (79.42%). This study divided participants’ personal monthly income into three groups. 1 means income is lower than 600 dollars, 2 equals income is between 600 dollars and 1200 dollars, and 3 equals income is over 1200 dollars. There was significant difference in their personal monthly income (F = 51.78, *p* < 0.000). This study also divided participants’ family monthly income into three groups. 1 means income is lower than 800 dollars, 2 equals income is between 800 dollars and 1600 dollars, and 3 equals income is over 1600 dollars. There was significant difference in their family monthly income (F = 54.833, *p* < 0.000).

**Table 1:** Sample Characteristics of Floating Population Covered by Different Medical Insurance

Variables	MIUE(*N* = 6095)	Other Inurances(*N* = 3924)	No Insurance(*N* = 1981)	Overall(*N* = 12,000)
	Mean (SD) or %	Mean (SD) or %	Mean (SD) or %	Mean (SD) or %
Age**, in years	33.82 (9.58)	32.02 (8.18)	32.09 (9.58)	32.95 (9.19)
Gender				
Male	57.29%	60.12%	55.83%	57.98%
Female	42.71%	39.88%	44.17%	42.03%
Education level				
Primary school or less	17.51%	5.28%	17.01%	13.43%
High school	76.19%	61.72%	74.56%	71.19%
More than high school	6.30%	33.00%	8.43%	15.38%
Marriage				
Spinsterhood	17.10%	22.83%	26.86%	20.58%
Married	82.90%	77.17%	73.14%	79.42%
Floating Area				
Interprovincial	70.75%	58.23%	73.35%	67.08%
Inside province	29.25%	41.77%	26.65%	32.92%
Occupation				
Civil servant	0.36%	3.59%	0.30%	1.46%
Manufacturing industry	52.67%	30.81%	56.60%	45.74%
Business & Services	44.98%	64.70%	40.62%	51.11%
Unemployed	1.99%	0.90%	2.48%	1.69%
Personal monthly income**, (1–3)				
lower than 600 dollars	50.66%	40.59%	54.78%	47.84%
between 600 dollars and 1200 dollars	37.86%	45.86%	34.91%	40.15%
over 1200 dollars	11.78%	13.55%	10.31%	12.01%
Family monthly income**, (1–3)				
lower than 800 dollars	35.64%	31.47%	44.78%	35.78%
between 800 dollars and 1600 dollars,	51.50%	50.54%	43.97%	49.94%
over 1600 dollars	12.86%	17.99%	11.25%	14.28%

**: *p* < 0.001

### Hospitalized health service utilization for floating population in Jiangsu province

#### Selection of inpatient facilities

**Fig. 1:**
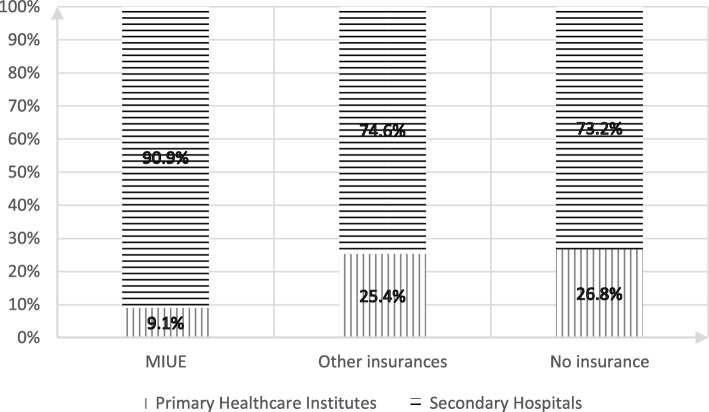
Table of Inpatient Facilities Selection for Floating Population in Jiangsu Province

The floating population of MIUE chose to be hospitalized in secondary hospitals and medical institutions which were above secondary levels (89.95%). It was higher than other medical insurances (74.76%) and no medical insurance (67.57%) by 15.19 and 22.38 percentage points respectively. By chi-square test (chi-square = 24.958, *p* = 0.000), *P*-value shows there is significant difference in Inpatient Facilities Selection for floating population with different medical insurance (Fig. 1).

#### In-hospital medical expenses

**Fig. 2:**
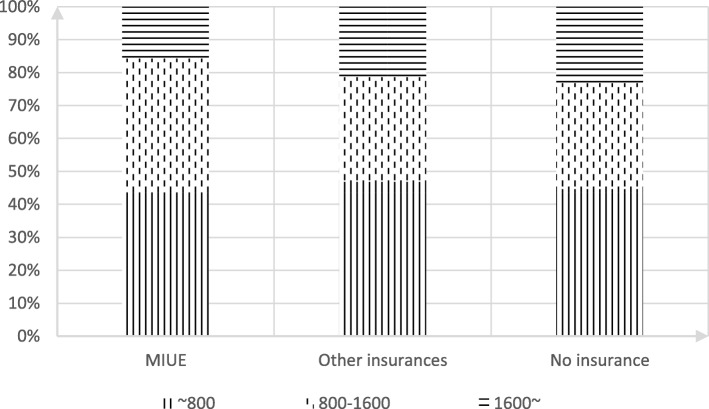
Hospitalization Expenses of Floating Population in Different Medical Insurance

Among the floating population investigated, the hospitalization expense which were within 800 dollars accounted for 51.80%. Those which were between 800 dollars and 1600 dollars accounted for 31.50%, and those which were above 1600 dollars accounted for 16.70%. Among them, 15.34% of the floating population with the medical insurance of urban employees have the hospitalization cost over 1600 dollars, which is lower than the floating population with other medical insurances (16.19%) and no medical insurance (21.62%) respectively by 0.85 percentage points and 6.28 percentage points. Chi-square test result (chi-square = 10.000, *p* = 0.040) shows the existence of significant difference between in-hospital medical expenses of the floating population covered by different medical insurances (Fig. 2).

#### Reimbursement of hospital expenses

**Fig. 3:**
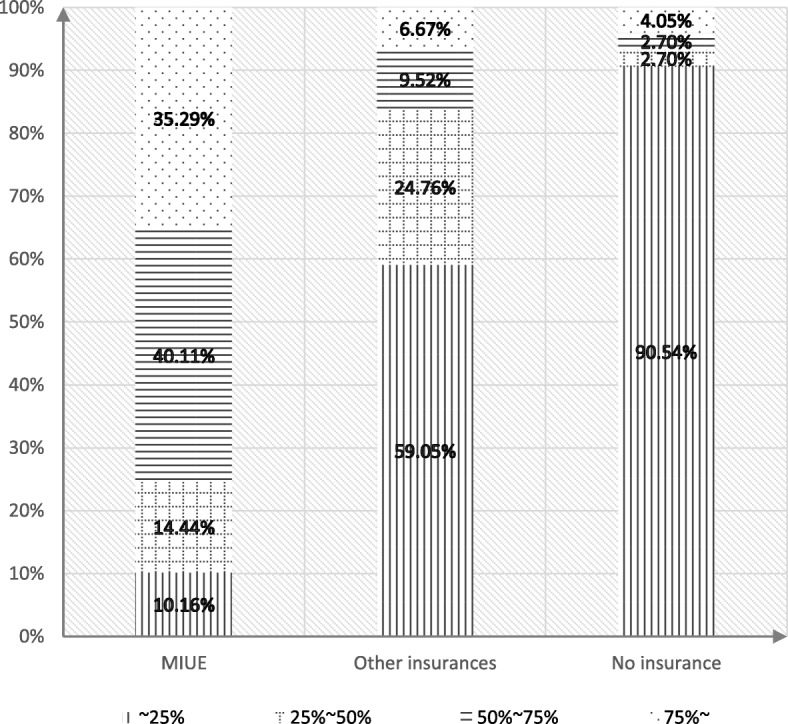
Reimbursement Ratio of Hospital expenses of Floating Population of Different Medical Insurance

The floating population with a history of hospitalization were surveyed within one year, and they were classified by the quartile of the reimbursement ratio. Fig. 3 shows that among the floating population who participated in the medical insurance for urban employees, 35.29% of them claimed the reimbursement ratio of hospitalization expenses was more than 75%, and only 10.16% of them claimed the ratio was less than 25%.

Among the floating population who participated in other medical insurances, 6.67% of them claimed the proportion of hospitalization expense reimbursement exceeded 75%. 59.05% of them claimed the reimbursement proportion was less than 25%.

Among the floating population without medical insurance, only 4.05% of them could get the reimbursement of medical expense exceeding 75%. While 90.54% of them could only get less than 25% reimbursement of hospitalization expense. By chi-square test (chi-square = 225.206, *p* = 0.000), significant difference exists between different medical insurance of floating population proportion and hospitalization medical expenses (Fig. 3).

**Fig. 4:**
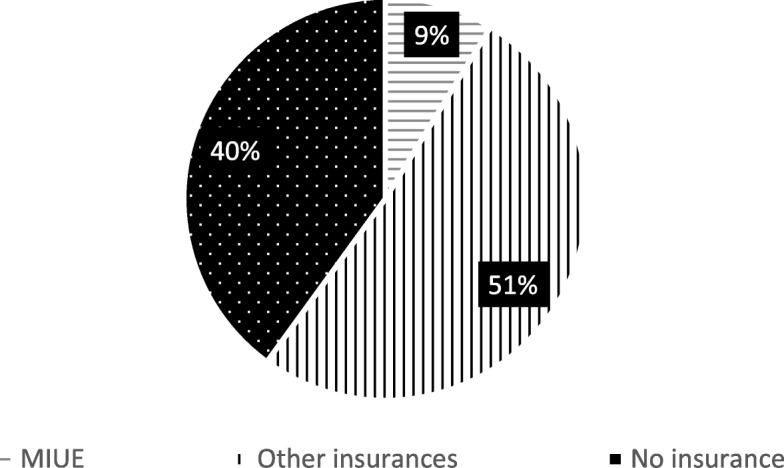
Analysis of the Hospital Expense Reimbursement Ratio of Floating Population with Different Medical Insurance is 0

One hundred sixty floating people did not receive any reimbursement. Only 9% of them had participated in MIUE. Participating in MIUE had seriously affected the hospitalization expenses reimbursement for floating population (Fig. 4).

### Factor analysis

#### Factor adaptability analysis

Bartlett’s spherical test results shows that chi-square = 12,822.19, *P* < 0.001 which indicates that the basic situation of the floating population is suitable for factor analysis. The KMO test result is 0.556 which indicates that the factor analysis is well adapted.

### Common factor extraction and function construction

After extracting the initial common factor by principal component method, the initial common factor is rotated by the maximum variance method. The eigenvalues of the first five common factors are all greater than 1. And the cumulative contribution of variance is 68.45% which reflects most of the information of 10 variables. According to the Rotational component matrix (Table 2), this study names the two factors (Age and Marital status) whose data are greater than 0.45 as “Demography”, names Personal monthly income and Family monthly income as “Income status”, and names Education, Hukou, Insurance status and Work status as “Social structure”. The one explained by factor 4 is “Gender” and the one explained by factor 5 is “Floating area”.

**Table 2:** Rotational component matrix of the influencing factors of HHSU in floating population

Variable Number	Variable Content	factor 1	factor 2	factor 3	factor 4	factor 5
		Demography	Income status	Social structure	Gender	Floating area
1	Age	0.831	−0.045	−0.081	− 0.059	0.164
2	Gender	0.087	−0.457	0.248	0.482	−0.543
3	Education	−0.514	0.305	0.47	0.178	0.221
4	Hukou	− 0.047	0.127	0.685	0.134	0.207
5	Marital status	0.784	0.276	0.023	0.11	−0.049
6	Floating area	0.109	−0.141	0.234	0.239	0.746
7	Occupation	0.023	− 0.071	0.075	−0.742	− 0.13
8	Personal monthly income	−0.018	0.851	0.031	−0.048	0.045
9	Family monthly income	0.332	0.696	0.169	0.264	−0.238
10	Insurance status	−0.03	−0.078	0.703	−0.352	− 0.115

After the common factor is extracted and the standardized factor score is obtained, the rotated variance contribution rate (C) is used as the weight coefficient to construct a comprehensive factor score function:

$$ F=0.17025\times {F}_1+0.16354\times {F}_2+0.13435\times {F}_3+0.11007\times {F}_4+0.10627\times {F}_5. $$

### Analysis of factors influencing the HHSU of floating population

There are four indicators reflecting the hospitalized health service utilization which includes Should be hospitalized but not, Inpatient facilities selection, In-hospital medical cost and Reimbursement of in-hospital medical cost.

#### Logistic regression of influencing factors of should be hospitalized but not

Logistic regression was conducted to investigate the influencing factors of Should be hospitalized but not. In this study, “Should be hospitalized but not” is categorical variable.

This study sets Factor 1(Demography), Factor 2(Income status), Factor 3(Social structure), Factor 4(Gender) and Factor 5(Floating area) as independent variables. And it also sets Inpatient facilities selection as dependent variable. According to β and *P*-values in Table 3, the Logistic regression results showed that “Social structure” had impacted “Should be hospitalized but not” of floating population in Jiangsu ;. In other words, floating population with worse medical insurance, Hukou, Education or Occupation was more likely to give up inpatient services when they need to be hospitalized. (Only statistically significant influencing factors analysis results are listed in the Table 3.)

**Table 3:** Logistic regression results of the influencing factors of should be hospitalized but not

Variables	Should be hospitalized but not	
	β	P-value
Demography(F1)	/	/
Income status(F2)	/	/
Social structure(F3)	−0.379	0.004
Gender(F4)	/	/
Floating area(F5)	/	/

#### Logistic regression of influencing factors of inpatient facilities selection

Logistic regression was conducted to investigate the influencing factors of Inpatient facilities selection. In this study, “Inpatient facilities selection” is categorical variable.

This study sets “Age, Gender, Education level, Hukou, Marriage, Floating area, Occupation, Insurance type, Personal monthly income and Family monthly income” as independent variables and Inpatient facilities selection as dependent variable.

Results in Table 4 shows that only Education level has been significantly associated with Inpatient facilities selection.

**Table 4:** Logistic regression results of Inpatient facilities selection

Variables	β	S.E,	P-value	OR	95% C.I. of OR	
					Lower limit	Upper limit
Age	0.322	0.301	0.286	1.379	0.764	2.489
Gender	−0.200	0.423	0.636	0.819	0.357	1.875
Education level	0.920	0.319	0.004	2.508	1.343	4.686
Hukou	0.690	0.524	0.188	1.994	0.714	5.566
Marital status	0.380	0.599	0.525	1.463	0.452	4.731
Floating area	−0.132	0.310	0.671	0.877	0.477	1.610
Occupation	−0.461	0.273	0.091	0.631	0.370	1.076
Insurance type	−0.317	0.234	0.176	0.729	0.460	1.153
Personal monthly income	0.169	0.275	0.539	1.184	0.691	2.029
Family monthly income	−0.153	0.282	0.589	0.859	0.494	1.492

#### Stepwise multiple linear regression of the influencing factors

Stepwise multiple linear regression was applied to explore the influencing factors of Inpatient facilities selection, In-hospital medical cost and Reimbursement of in-hospital medical cost. This study sets Factor 1, Factor 2, Factor 3, Factor 4 and Factor 5 as independent variables, and sets In-hospital medical cost and Reimbursement of in-hospital medical cost as dependent variables.

Table 5 presents the results of the stepwise multiple linear regression which demonstrated that Factor 1, Factor 3, Factor 4 and Factor 5 were statistically associated with the influencing factors, including In-hospital medical cost and Reimbursement of in-hospital medical cost. According to the coefficients and *P*-values, significant differences were observed among different influencing factors:

**Table 5:** Multiple linear regression results of factors influencing the HHSU floating population

Variables	In-hospital medical cost		Reimbursement of in-hospital medical cost	
	β	*P*-value	β	*P*-value
Demography(F1)	872.689	0.000	/	/
Income status(F2)	/	/	/	/
Social structure(F3)	/	/	0.134	0.000
Gender(F4)	/	/	−0.099	0.001
Floating area(F5)	526.253	0.007	/	/

- Factor 1 “Demography” had significant impact on In-hospital medical cost, which indicated that older inpatients have cost more in the hospitalized health service utilization.
- Factor 3 “Social structure” had great impact on Reimbursement of in-hospital medical cost. When the inpatients had a higher social status, such as higher education, better Hukou and better occupation, they could get higher reimbursement of in-hospital medical cost and choose higher level medical facilities.
- Factor 4 “Gender” had great impact on Reimbursement of in-hospital medical cost, which reflected male inpatients were more inclined to get higher reimbursement.
- Factor 5 “Floating area” had significant impact on In-hospital medical cost. The floating population who had registered outside of Jiangsu province but now lived in Jiangsu province had spent more money in hospitalized health service utilization.

## Discussion

### The medical insurance situation has significant influence on inpatient facilities selection

The data of this study shows that the proportion of floating population choosing the hospitals at the county level and below was 69.0% which was lower than the fifth national health service survey (72.6%). Different basic medical insurance has significant influence on the choice of hospital institutions (chi-square = 24.958, *p* = 0.000). This phenomenon is widespread, and similar findings are often found in domestic studies about floating population: Wang found that the proportion of floating population in hospitals at county level and below is relatively low which is only 56.4% [17]. Floating population struggled to benefit from health insurance; Zheng used t test to find that there was a difference in the selection of hospitalization institutions for people with different medical insurances (*p* = 0.000, 95%CI = 1.07–1.50) [18].

There are many reasons of this result from many aspects. Domestic scholars generally believed that the main reason was the floating population’s choice of medical institutions was restricted by the level of medical services and their own income.

First of all, the floating population would choose to buy their own medicine or resistance for treatment if they had diseases [19]. The reimbursement ratio of urban workers’ medical insurance is relatively large. Without substantial reimbursement, the floating population who did not participate in urban workers’ medical insurance may thought that the consumption of formal medical institutions was too high to afford [20].

In addition, floating population would obtain different reimbursement proportion for hospitalization at different levels medical institutions. Under general circumstance, different medical insurances have different reimbursement lines and reimbursement scale. Floating population without MIUE would not choose to go to high level hospitals.

The location of hospitals also influences floating population choosing HHSU and it is a significant factor [21]. It also reduces the fairness of health services among hospitals.

### Medical insurance has a significant impact on reimbursement of in-hospital medical cost

As mentioned above, there is a significant difference in the proportion of hospitalization expenses reimbursement for the floating population who have participated in different medical insurances. And more than 30% (33.97%) of the inpatients did not receive any reimbursement. They paid their expense all by themselves. Among them, 40% of the patients participated in the basic medical insurance other than the medical insurance for urban employees, but failed to get any reimbursement. In this case, other basic medical insurances that the patients participated in completely lost the role of risk sharing. Yao Yi found that although they all belonged to the basic medical system, the benefits of each system were unfair to some extents. The expense coverage of MIUE was significantly higher than new rural cooperative medical system. So the low-income families would face high hospitalization expense burden [22].

First, HHSU’s reimbursement is restricted by geography. China’s basic medical insurance does not have a unified regulation of long-distance medical treatment. It does not reach a consensus. And various pooling areas have their own closed management [23–25]. As the development of each pooling region is different, the pooling fund, reimbursement scope and standard are also very different. So the medical insurance policies issued by different regions are different [26–28]. As stipulated by the regional medical insurance policy, only the hospitalization within the region under the jurisdiction can be qualified for reimbursement [25, 28, 29]. According to the regulations of some regions, floating population cannot enjoy the same reimbursement ratio as the local resident population, nor can they enjoy the same reimbursement ratio when they return home to be hospitalized. Secondly, while the reimbursement ratio is low, the reimbursement procedures and process are very tedious and the compensation lags behind [25]. The vast majority of rural farmers in China still apply for reimbursement for medical treatment in different places. There is a situation that inpatients pay the bills for the moment and the bills are sent back or taken back to the insured places for reimbursement. When reimbursement is being processed, relevant evidences such as medical records, inspection sheets, expense lists, medical advice and discharge instructions should be provided. The procedures are complex and complicated, and the floating population have to shuttle back and forth between two places. It will greatly increase the personal burden and time waste. It also will seriously affect the implementation of medical insurance treatment [28, 30].

### The type of medical insurance has a significant impact on the utilization of hospital health services for floating population

The type of medical insurance has a significant impact on whether they should be hospitalized or not. The utilization of medical service of floating population is seriously inadequate, and the level of medical security is the main factor influencing the utilization of medical service towards floating population.

Some scholars believe that the low coverage and low level of medical security of floating population make the great majority of floating population lack necessary medical security. Coupled with the high medical costs in cities, floating population bears a huge burden of disease economy. These factors hinder the use of hospitalized medical services towards floating population [31, 32]. The results of this study believed that different types of medical insurance affect the utilization of hospitalized health services of floating population [33]. Gao’s research shows that the accessibility of hospitalization services for urban residents, new rural cooperative residents and urban employees who have participated in the insurance has decreased in turn [34]. But this is different from the results of this study. In this study, the floating population who participate in MIUE have the Should be hospitalized but not hospitalized condition slightly less than other basic medical insurance types.

There are many reasons for this result. For example, due to the limitation of medical insurance, the primary factor that floating population will consider is the cost of the medical service and their own income situation. The floating population will pay more attention to the economic factors of hospitalization health service. Medical insurance has a great impact on the utilization of hospital health services of floating population [35]. Under its influence, the utilization of hospital health services of floating population in Jiangsu province is not optimistic, especially the floating population who participate in other insurances and who has no medical insurance. The type of medical insurance has a certain influence on the health condition of the floating population. In order to reduce the proportion of floating population who have not been hospitalized due to economic difficulties, health education can be carried out to provide targeted [36–38], considerate and more practical services for floating population. Besides, we should also make floating population be aware of protecting their health [24, 39]. At the same time, it is necessary to increase the reimbursement ratio of medical insurance for urban and rural residents [24, 40], and start to make the basic medical insurance cover the whole population, help floating population with financial difficulties from the perspective of medical insurance [28].

## Conclusion

All in all, there is a gap between the hospitalized health service utilization in floating population covered by different medical insurances.

Although medical insurances provide the most basic health protection function, the results of this study show that the medical insurance of the floating population in Jiangsu Province has lost its risk sharing role. The unfair distribution of inpatient medical services, high costs, the pessimistic reimbursement of medical insurance and the floating population participating in different medical insurances are still influencing the proceeding of fair inpatient medical services.

Increasing the education of health knowledge for the floating population, guiding them in an orderly manner to rationally select inpatient medical facilities, and helping them understand the medical insurance policy can help them participate in more appropriate medical insurances, get more professional medical services, and obtain more reimbursement they deserved. The government should strengthen its concern for the floating population and set up special medical insurance for the floating population to achieve the goal of reducing the hospitalization burden of the floating population.

## Data Availability

This study was a secondary analysis of de-identified data. The “National Internal Migrant Dynamic Monitoring Survey, 2014” data is publicly available to authorized researchers who have been given permission by the National Population and Family Planning Commission. The data that support the findings of this study are available from National Population and Family Planning Commission but restrictions apply to the availability of these data, which were used under license for the current study, and so are not publicly available. Data are however available from the authors upon reasonable request and with permission of National Population and Family Planning Commission.

## Abbreviations

HHSU: Hospitalized health service utilization
MIUE: Medical Insurance of Urban Employee
MIUR: Medical insurance for urban residents
MIURR: Medical insurance for urban and rural residents
NCMS: New rural cooperative medical system
NHFPC (DSMMP): Department of Services and Management for Migrant Population of National Health and Family Planning Commission of China
